# An ANCCA/PRO2000-miR-520a-E2F2 regulatory loop as a driving force for the development of hepatocellular carcinoma

**DOI:** 10.1038/oncsis.2016.22

**Published:** 2016-05-30

**Authors:** J Huang, J Yang, Y Lei, H Gao, T Wei, L Luo, F Zhang, H Chen, Q Zeng, L Guo

**Affiliations:** 1 Department of Pathology, Zhujiang Hospital, Southern Medical University, Guangdong, China; 2 Department of Dermatology, Zhujiang Hospital, Southern Medical University, Guangdong, China; 3 Department of Biochemistry and Molecular Medicine, University of California at Davis Cancer Center, Davis, CA, USA

## Abstract

Hepatocellular carcinoma (HCC) is one of the most common malignancies in Asia especially in China. We previously identified that ANCCA/PRO2000 as an important proliferation-associated protein predicted poor prognosis of patients with HCC. However, the molecular mechanisms of ANCCA/PRO2000 leading to hepatocarcinogenesis and progression are still obscure. In the present study, we found that ANCCA/PRO2000 overexpression in HCC specimens correlated with aggressive tumor behavior and poor survival. Furthermore, ANCCA/PRO2000 exerts strong oncogenic function in HCC and promotes cell proliferation by regulating E2F2 expression, a critical cell cycle regulator. Notably, miR-520a is an intermediate regulator between ANCCA/PRO2000 and E2F2. Mechanistically, ANCCA/PRO2000 not only interacts with E2F2 but also negatively regulates miR-520a that inhibits E2F2 to cooperatively promote *in vitro* and *in vivo* growth of HCC cells. Moreover, we demonstrated that ANCCA/PRO2000 enhances the migratory capacity of HCC cells partially by suppressing ERO1L and G3BP2 expression. Additional research identified that miR-372, as a prognostic factor for HCC, could directly target ANCCA/PRO2000. Our results suggest the ANCCA/PRO2000-miR-520a-E2F2 regulatory loop as a driving force for HCC development and ANCCA/PRO2000 as a potential therapeutic target for HCC.

## Introduction

Hepatocellular carcinoma (HCC) is the most common malignancy in Asia especially in China and the fifth most common worldwide, accounting for one million deaths annually.^1, 2^ Genetic alterations such as amplification, deletion, translocation and rearrangement have been proven to be the frequent events and may have the important roles in the pathogenesis of HCC. Any type of genetic alterations could result in gene aberrant expression either up- or downexpression and subsequently lead to abnormal biological aspects in cell proliferation, differentiation, motility and survival. Cytogenetic profiling studies have suggested that a subset of frequent gain of chromosomes 1q, 6p, 8q, 17q and 20q, and frequent loss of chromosomes 1p, 4q, 6q, 8p, 13, 16 and 17p.^3, 4^ Gain of chromosomes 1q and 8q has particularly been associated with the early development of HCC.^4^ In the region of chromosome 8q, PRO2000 was showed increased expression (4.7-fold) in a majority of HCC samples comparing with corresponding non-cancerous sample by comparative genomic microarray analysis. It suggested that *PRO2000* may be an important candidate gene that is located within a region of chromosome 8q in HCC.^5^

PRO2000, also named as AAA nuclear coregulator cancer-associated protein (ANCCA) or AAA domain containing 2, is a novel member in the superfamily of AAA+ (ATPases associated with various cellular activities) proteins.^6^ It shares the conserved region of ~220 amino acids that contains an ATP-binding site with the other members of ATPases family. The ANCCA/PRO2000 protein encoded by this gene contains two AAA+ ATPase domains (AAA-D1 and AAA-D2) and a bromodomain. AAA+ family proteins have critical roles in diverse biological processes via their ATPase-driven remodeling of macromolecular complexes.^7, 8, 9^ Several researchers have identified that ANCCA/PRO2000 functionally controls the crucial regulators of cell proliferation and survival pathways including E2F1/3, EZH2, B-Myb and MYC in breast cancer, prostate cancer, lung adenocarcinoma and endometrial cancer. ANCCA/PRO2000 is always overexpressed in cancer cells and correlates with poor overall survival and disease recurrence.^6, 10, 11, 12, 13, 14, 15, 16^ We previously identified that ANCCA/PRO2000 functions as an important proliferation-associated protein and a predictor of poor prognosis in HCC.^17^ Studies from others also disclose that ANCCA/PRO2000 with a high expression in HCC could regulate the proliferative, invasive and migratory capacity of HCC cells.^18, 19^ However, the molecular mechanism of ANCCA/PRO2000 in the hepatocarcinogenesis and progression remains unclear yet.

MicroRNAs (miRNAs) are small noncoding RNAs of ~22 nt with a role in post-transcriptional regulation of target messenger RNA (mRNA) translation by targeting 3′-untranslated region (UTR) of it. Increasing evidence has demonstrated that miRNAs have pivotal roles in multiple cellular processes such as cell proliferation, differentiation and apoptosis.^20, 21^ Moreover, the aberrant expression of miRNAs has been found in tumoriogenesis and progression of various cancers, including HCC, as novel classes of oncogenes and tumor suppressors by directly targeting many critical protein-coding genes.^22, 23, 24^ For example, miR-423 promotes cell growth and regulates G1/S transition by targeting p21Cip1/Wafl.^25^ MiR-124 inhibits the invasive and metastatic potential of HCC by its target on *ROCK2* and *EZH2* genes.^26^ Previous studies showed that miR-520a was associated with proliferation and progression of esophageal squamous cell carcinoma and non-small cell lung cancer,^27, 28^ but its function in HCC is still unknown. As key post-transcriptional modifiers, more and more miRNAs have been reported to orchestrate the regulation of cellular signaling networks by acting as critical intermediary regulators of a variety of crucial regulatory loops.^29, 30^ Characterization of these miRNA-mediated regulatory loops may be of great importance to comprehensively understand the molecular mechanisms contributing to the development of HCC.

In this study, we first investigated the biological function of ANCCA/PRO2000 expression in HCC, as a critical oncogenic factor.^31^ Next, we provided *in vitro* and *in vivo* evidence suggesting that ANCCA/PRO2000 could not only interact with E2F2 but also negatively regulate miR-520a that inhibits E2F2 to cooperatively promote cell proliferation and tumor xenograft growth of HCC. Moreover, we demonstrated that ANCCA/PRO2000 enhances the migratory capacity of HCC cells partially by suppressing endoplasmic reticulum oxidoreductin 1 (ERO1L) and Ras-GTPase-activating protein-SH3-domain-binding protein 2 (G3BP2) expression. Additional research identified that miR-372, as a prognostic factor for HCC, could directly target ANCCA/PRO2000.

## Results

### Expression of ANCCA/PRO2000 and its correlation with clinicopathological characteristics and prognosis in HCC

**Figure 1:**
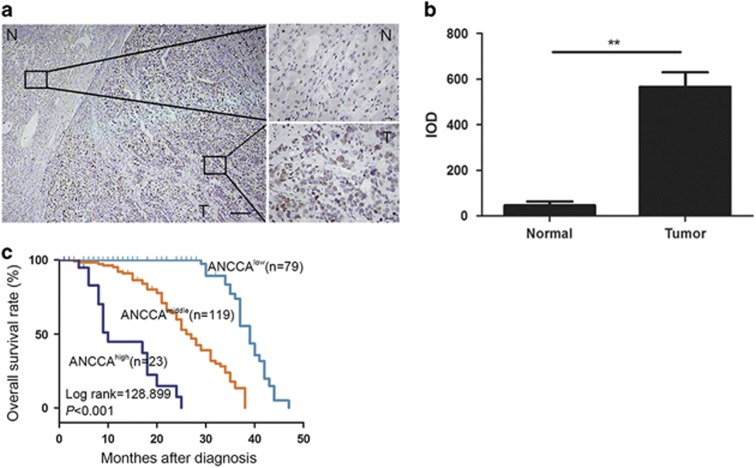
Aberrant expression of ANCCA/PRO2000 in HCC was correlated with poor patient survival. (**a**) Representative immunohistochemistry staining of ANCCA/PRO2000 in HCC (T) and adjacent nontumor tissues (N). Lower-magnification view was shown on the left, and higher-magnification views on the right. (**b**) ANCCA/PRO2000 expression assessment as integrated optical density scores. **P<0.01. *P*-values were determined using independent samples *T-*test. (**c**) Kaplan–Meier analysis of overall survival of 221 patients with HCC based on ANCCA/PRO2000 expression. −, negative; +, moderate positive; ++, strong positive. Scale bars, 100 μm (**a**, left); 20 μm (**a**, right).

To understand the clinicopathologic significance of ANCCA/PRO2000 in HCC tissue, a total of 221 HCC patients who had undergone tumor resection were analyzed by immunohistochemistry staining. ANCCA/PRO2000 expression was localized in nucleus of the cancer cells, whereas few scattered positive cells were detected in adjacent nontumor tissues (Figure 1a). The intensity of ANCCA/PRO2000 staining in cancer cells was much stronger than that in non-cancer cells (Figure 1b). Table 1 summarized the correlation between ANCCA/PRO2000 expression and clinicopathologic characteristics in HCC patients. By Pearson *χ*^2^-test, the results indicated that ANCCA/PRO2000 expression was significantly associated with the tumor size, tumor number, tumor differentiation, cirrhosis, tumor node metastasis (TNM) stages, venous metastasis, microsatellite tumors and the recurrence, whereas no significant differences were observed with respect to gender, age, serum HBsAg and serum alpha fetal protein (AFP) (Table 1). The Kaplan–Meier survival curves and univariate analysis revealed that high-level expression of ANCCA/PRO2000 was correlated with short overall survival of the HCC patients (*P*<0.001; Figure 1c, Table 2). Further multivariate analysis showed that ANCCA/PRO2000 was independent prognostic factor for HCC patients (Table 2).

### ANCCA can promote growth and invasion of HCC cells *in vitro*

**Figure 2:**
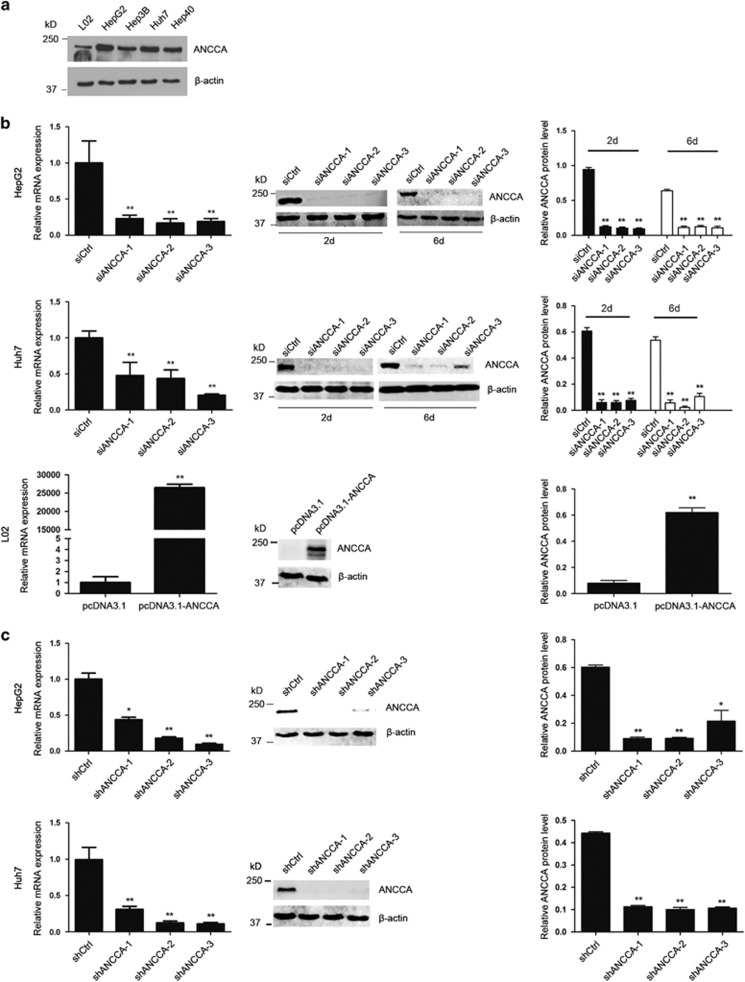
Expression of ANCCA/PRO2000 in liver cell lines. (**a**) Western blotting analysis of ANCCA/PRO2000 protein expression in liver cell lines L02, HepG2, Hep3B, Huh7 and Hep40. (**b**) qRT–PCR and western blotting analyses confirmed that downregulaiton of ANCCA/PRO2000 in HepG2 and Huh7 via siANCCA (100 pmol) and upregulation in L02 cells by pcDNA3.1-ANCCA (2 μg). (**c**) qRT–PCR and western blotting analyses of ANCCA/PRO2000 expression in HepG2 and Huh7 cells infected with lentiviral particals of shANCCA. Error bars indicate mean±s.d. from three independent experiments. *P<0.05; **P<0.01. *P*-values were determined using independent samples *T-*test.

The significant elevated expression of ANCCA/PRO2000 in HCC tissues prompted us to further investigate its effect on proliferation of HCC cells. Western blotting revealed that HCC cell lines showed significantly elevated levels of ANCCA/PRO2000 compared with normal liver cell line L02. Moreover, HepG2 and Huh7 cells appeared to express higher levels of ANCCA/PRO2000 than Hep3B and Hep40 cells (Figure 2a). Consequently, L02, HepG2 and Huh7 cell lines were chosen to be further studied in the following research.

Next, small interfering RNAs (siRNAs) targeting to ANCCA/PRO2000 (siANCCA-1, siANCCA-2 and siANCCA-3) or short hairpin RNAs targeting to ANCCA/PRO2000 (shNCCA-1, shANCCA-2 and shANCCA-3) were used to suppress ANCCA/PRO2000 expression in HepG2 and Huh7 cells. ANCCA/PRO2000 overexpression plasmid (pcDNA3.1-ANCCA) was transfected into L02 cells to upregulate ANCCA/PRO2000 expression. Quantitative reverse-transcription PCR (qRT–PCR) and western blotting analyses confirmed the effect of siANCCA, shANCCA and pcDNA3.1-ANCCA (Figures 2b and c).

**Figure 3:**
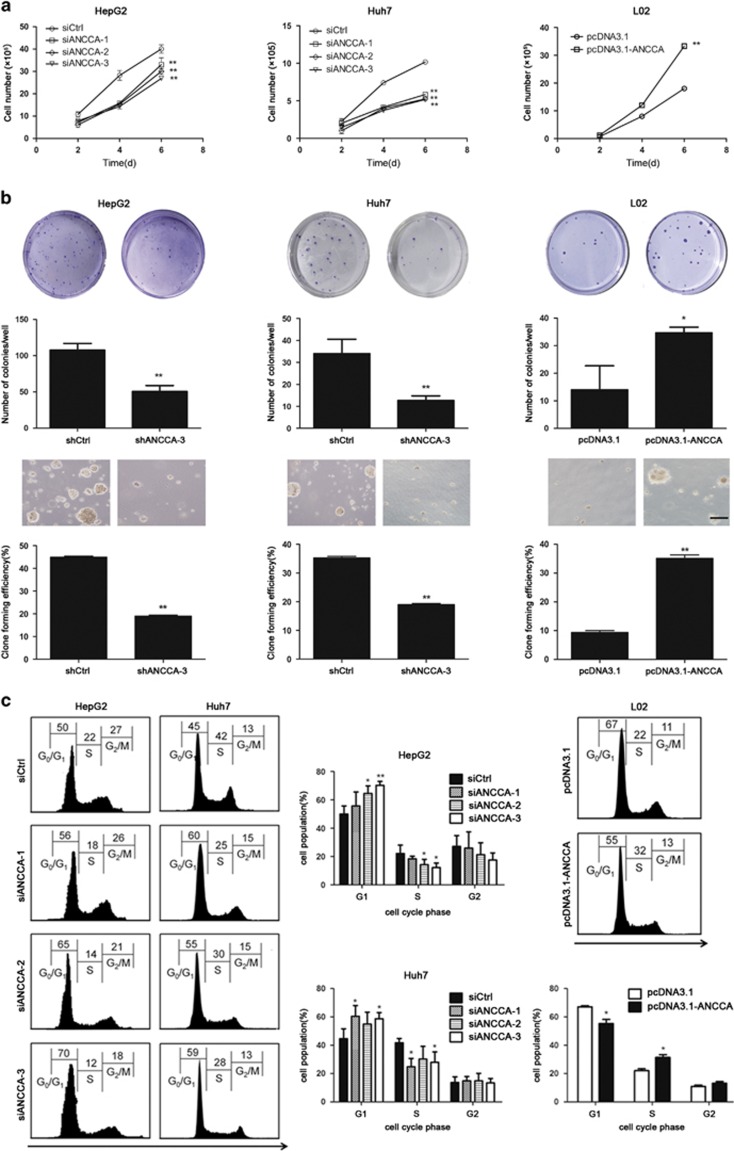
ANCCA/PRO2000 affected cell proliferation *in vitro* by interfering with cell cycle progression. (**a**) Proliferation assays of HepG2, Huh7 and L02 cells with altered ANCCA/PRO2000 expression. Each sample was assayed in triplicates for 6 consecutive days. (**b**) Plate clone-forming (upper) and soft-agar colony (lower) assays of HepG2, Huh7 and L02 cells. Representative photographs are shown, and the numbers of colonies were counted. Scale bar, 100 μm. (**c**) FACS analysis of distribution of cells in each cell cycle phase. Representative FACS profiles were shown, on which numbers indicate percentage of cells in G0/G1, S or G2/M phase. Error bars indicate mean±s.d. from three independent experiments. *P<0.05; **P<0.01. *P*-values were determined using independent samples *T-*test.

Then, CCK-8 and colony formation assays were performed to measure cell proliferation and growth. As shown in Figure 3a, the viability of HepG2 and Huh7 cells transfected with siANCCA significantly decreased compared with that of control-siRNA (siCtrl)-treated cells, whereas upregulation of ANCCA/PRO2000 in L02 cells markedly promoted cell growth. Furthermore, a statistically significant decrease in colony formation was observed between siANCCA-treated cells and the controls, and the number of colonies increased in ANCCA/PRO2000 overexpression L02 cells (Figure 3b). Flow cytometry analysis found that both HepG2 and Huh7 cells showed increased percentages in the G0–G1 phase after ANCCA/PRO2000 downregulation, which paralleled with decreased percentages in the S-phase. Consistently, ANCCA/PRO2000 overexpression prevented cell cycle arrest in L02 cells with a higher percentage in S-phase and a lower percentage in G0–G1 phase (Figure 3c). These data suggested that ANCCA/PRO2000 affected the liver cancer cell proliferation by interfering with the cell cycle progression.

**Figure 4:**
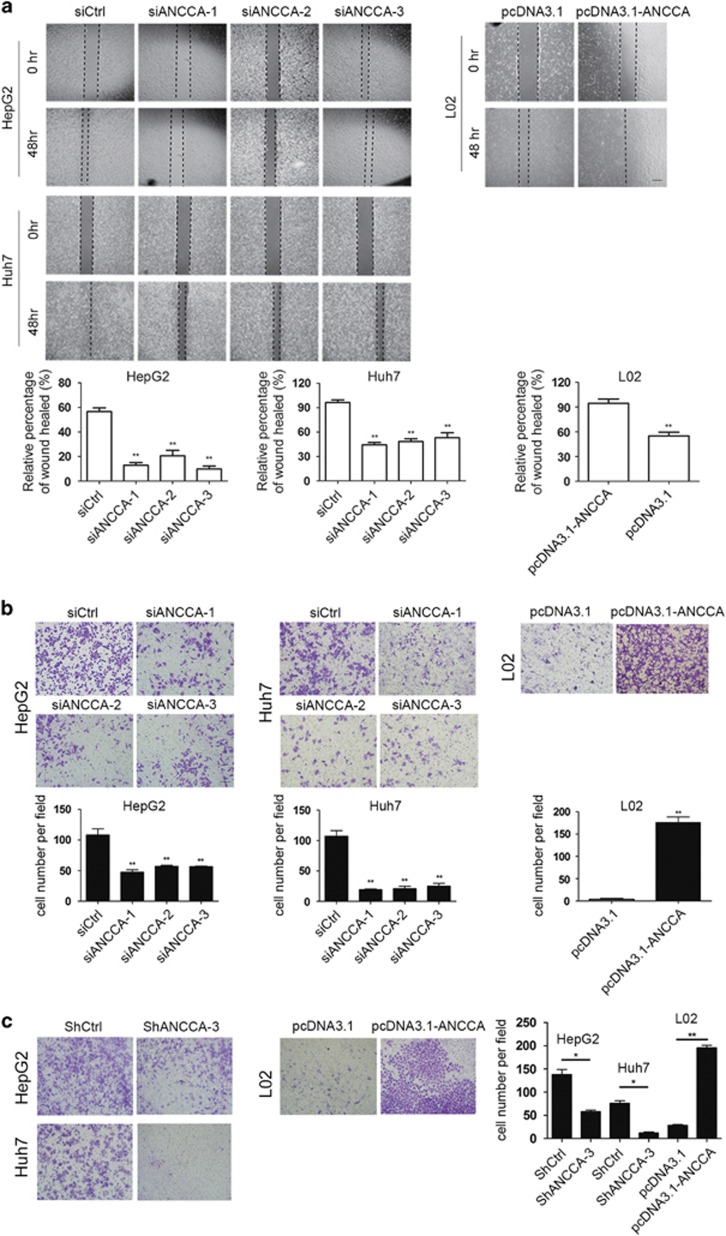
ANCCA/PRO2000 promoted the cell migration and invasiveness of HCC cells *in vitro*. (**a**) Cell migration was quantified by wound-healing assay. Cells were imaged immediately (0 h) and 48 h after scratches were created to measure the percentage of wound-healed area. Representative images at different time points are shown. Scale bar, 150 μm. (**b**, **c**) Transwell cell migration assay (**b**) and Matrigel invasion assay (**c**). Representative photographs are showed in the upper panels. Quantitative measurements are showed in the lower panels. Data represent mean±s.d. of three independent experiments. Scale bars, 100 μm. *P<0.05; **P<0.01.

We further investigated whether ANCCA/PRO2000 was required for migratory and invasive capabilities. The wound-healing and transwell migration assay showed that ANCCA/PRO2000 knockdown attenuated cell motility in HepG2 and Huh7 cells compared with that in siCtrl-treated cells (Figures 4a and b). Similarly, the matrigel invasion assay demonstrated that the invasiveness of shANCCA-treated HepG2 and Huh7 cells was significantly lower (Figure 4c). Meanwhile, the opposite results were also observed in ANCCA/PRO2000 overexpressing L02 cells (Figure 4). Taken together, both loss- and gain-of-function experiments demonstrated that ANCCA/PRO2000 promoted the cell migration and invasiveness of HCC cells *in vitro*.

### ANCCA/PRO2000 enhances tumorigenicity of HCC cells *in vivo*

**Figure 5:**
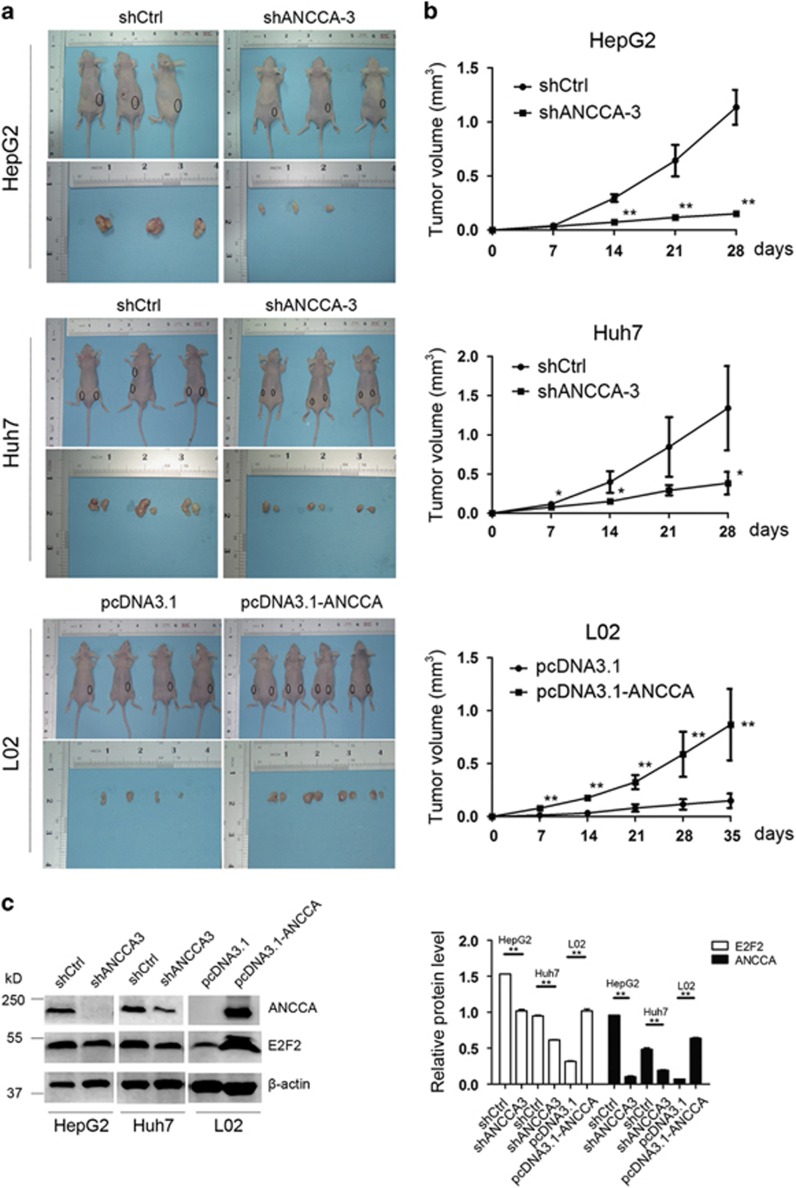
ANCCA/PRO2000 enhanced tumorigenicity of HCC cells in nude mice. (**a**) Tumors formation of cells stably with either high or low ANCCA/PRO2000 expression (*N*=3 or 4 mice for each group). (**b**) Growth curve of tumor volumes. (**c**) Western blotting analysis of ANCCA/PRO2000 and E2F2 protein expressions in tumor xenografts. The error bars represent mean±s.d. *P<0.05; **P<0.01, versus shCtrl or pcDNA3.1.

The tumorigenic properties of ANCCA/PRO2000 *in vivo* were conducted in nude mouse xenograft. Figures 5a and b showed the subcutaneous tumor growth of HepG2 and Huh7 infected with shANCCA or control-short hairpin RNA (shCtrl). The tumor growth was significantly slower in nude mice inoculated with shANCCA-infected HepG2 and Huh7 cells as compared with the vector control mice. In agreement with the tumor growth curve, the average tumor volumes for shANCCA-infected HepG2 and Huh7 cells were significantly smaller than those of the control cells. Furthermore, ANCCA/PRO2000 overexpression L02 cells showed accelerated tumor formation and significantly larger tumor size compared with the control cells. Therefore, we concluded that ANCCA/PRO2000 served as a potential oncogene of HCC both *in vitro* and *in vivo.*

### Identification of ANCCA/PRO2000-associated genes to control cell growth of liver cancer cells

**Figure 6:**
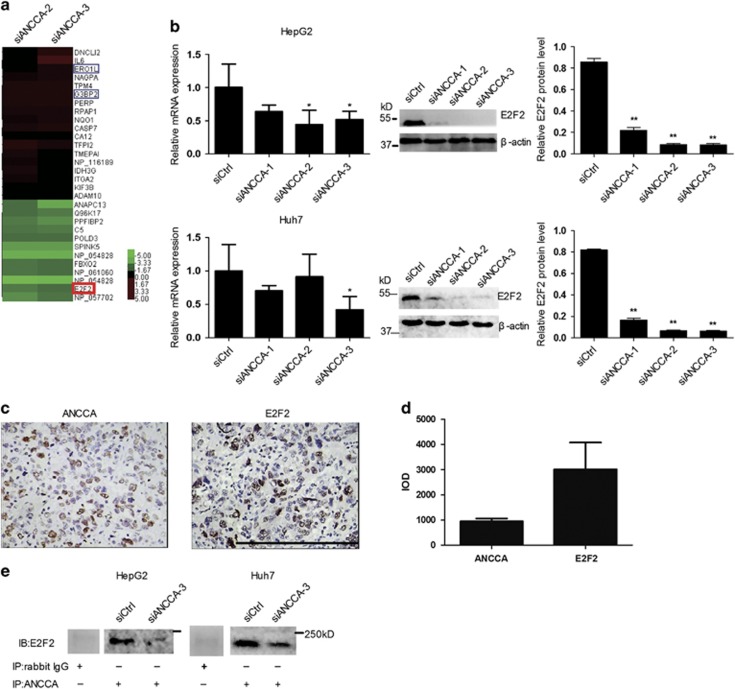
Identification of ANCCA/PRO2000-associated genes to control cell growth of liver cancer cells. (**a**) Unsupervised hierarchical cluster analysis of differentially expressed genes screened by cDNA microarray. Rows, biological samples; columns, mRNAs; red, expression value higher than average expression across all samples; blue, expression value lower than average. E2F2 is indicated by red box, and ERO1L and G3BP2 are indicated by blue box. (**b**) qRT–PCR and western blotting validated significant inhibition of E2F2 expression in siANCCA-treated cells. Data represent mean±s.d. of three independent experiments. (**c**, **d**) Representative immunohistochemistry staining and intensity of ANCCA/PRO2000 and E2F2 in HCC tissues. Scale bars, 200 μm. (**e**) The association between ANCCA and E2F2 was analyzed by co-immunoprecipitation in HepG2 and Huh7 cells treated with siANCCA or siCtrl. *P<0.05; **P<0.01 versus siCtrl.

To identify molecular targets associated with ANCCA/PRO2000, a cDNA microarray analysis was applied to HepG2 cells transected with siANCCA. Twelve genes were downregulated and 25 genes were upregulated for more than twofold in siANCCA-treated cells (Figure 6a). Among these genes, *E2F2* that was downregulated for 2.58-fold was the only one cell cycle-related gene with ANCCA/PRO2000 depletion. On the basis of the microarray data, qRT–PCR and western blotting analysis were performed to confirm whether *E2F2* was ANCCA/PRO2000-associated gene in liver cancer cells. When compared with siCtrl-treated cells, siANCCA-treated cells showed a modest decrease of E2F2 at both mRNA and protein levels in HepG2 and Huh7 cells (Figure 6b).

In agreement with the results observed upon ANCCA/PRO2000 knockdown *in vitro*, western blotting revealed a marked decrease of E2F2 in shANCCA-treated cells when compared with shCtrl-treated cells in mouse xenograft tumors (Figure 5c). Moreover, we analyzed ANCCA/PRO2000 and E2F2 protein expression in HCC tissue using immunohistochemistry. E2F2 expression is positively correlated with ANCCA/PRO2000 in cancer cells (Figures 6c and d).

The above data provided evidence that ANCCA/PRO2000 may be closely associated with E2F2. Further co-immunoprecipitation (co-IP) showed that ANCCA/PRO2000 could interact E2F2 through protein–protein binding. (Figure 6e). Moreover, E2F2 was accompanied with a robust decrease of ANCCA/PRO2000 in siANCCA-treated HepG2 and Huh7 cells. The above results indicated that ANCCA/PRO2000 regulates E2F2 expression at both mRNA and protein levels.

### miR-520a is an intermediate regulator between ANCCA/PRO2000 and E2F2

**Figure 7:**
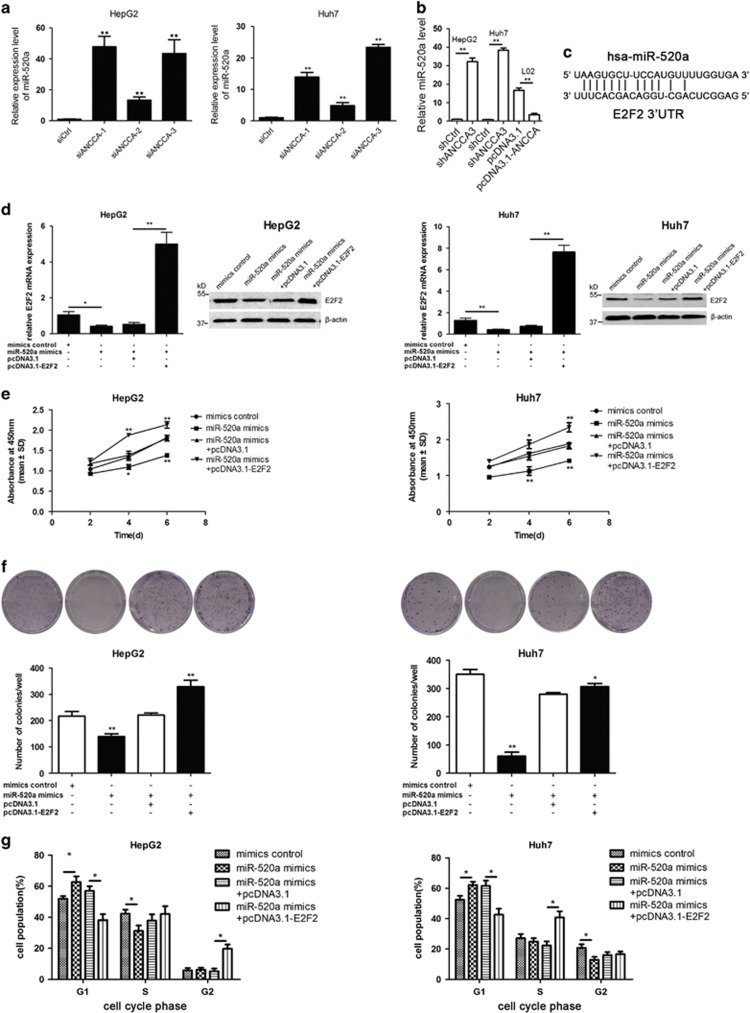
miR-520a is an intermediate regulator between ANCCA/PRO2000 and E2F2. (**a**) ANCCA/PRO2000 inversely regulates miR-520a expression in HepG2 and Huh7 cells. (**b**) qRT–PCR analysis of miR-520a expression in tumor xenografts. (**c**) Predicted binding sites of miR-520a in E2F2 3′-UTR. (**d**) E2F2 expression was analyzed by western blotting in HepG2 and Huh7 cells after transfection with miR-520a mimics alone, the combination of miR-520a mimics and pcDNA3.1-E2F2, or the corresponding controls. (**e**) Cell growth curves of HepG2 and Huh7 cells. (**f**) Plate clone-forming assay of HepG2 and Huh7 cells. Representative photographs are shown, and the numbers of colonies were counted. (**g**) FACS analysis of distribution of cells in each cell cycle phase. Data are shown as mean±s.d. derived from three independent experiments. *P<0.05; **P<0.01, versus siCtrl, mimics control or miR-520a mimics+pcDNA3.1.

A previous literature suggested that miR-520a was barely expressed and could suppress cell growth in HCC.^32^ Then, we further tested whether ANCCA/PRO2000 could modulate miR-520a by measuring miR-520a expression after silencing ANCCA/PRO2000 in HCC cells. Results showed that miR-520a expression was noticeably elevated when ANCCA was silenced with siRNAs (Figure 7a). Consistent with the finding *in vitro*, qRT–PCR analysis showed a marked increase of miR-520a expression in shANCCA-treated cells as compared with that in shCtrl-treated cells in xenograft tumors (Figure 7b).

As miRNAs can regulate their target gene expression, a bioinformatics search for putative miR-target gene pairs was conducted. Interestingly, both of two independent prediction programs (TarScan and miRanda) predicted E2F2 as a putative target of miR-520a because there is an exact match between the E2F2 3′-UTR and miR-520a (Figure 7c). To determine whether miR-520a can modulate E2F2 expression, we transfected HepG2 and Huh7 cells with miR-520a mimics or mimics control. Results showed that E2F2 expression was significantly reduced at both mRNA and protein levels after treatment with miR-520a mimics (Figure 7d), suggesting that miR-520a could inhibit E2F2 expression.

Next, we performed CCK-8 and plate clone-forming assays to confirm whether miR-520a repressed cell growth in HCC as previously reported. As shown in Figures 7e and f, the viability of HepG2 and Huh7 cells transfected with miR-520a mimics was significantly impaired compared with that of mimics control-treated cells, and the number of colonies also decreased after the introduction of miR-520a mimics. Furthermore, flow cytometry was employed to analyze cell cycle distribution. The percentages of HepG2 and Huh7 cells exposed to miR-520a mimics in G0–G1 phase increased compared with that of mimics control-treated cells, which was accompanied with a decrease of that in S- or G2-phase (Figure 7g). These data indicated that miR-520a suppressed cell growth in HCC by interfering with the cell cycle progression.

To further explore whether miR-520a affect cell growth in an E2F2-dependent manner, we transfected E2F2 overexpression plasmid (pcDNA3.1-E2F2) or control plasmid (pcDNA3.1) into miR-520a mimics-treated HCC cells. qRT–PCR and western blotting assays showed that E2F2 expression was noticeably elevated in HCC cells co-transfected miR-520a mimics and pcDNA3.1-E2F2 (Figure 7d). Moreover, as evidenced by the results in Figures 7e–g, E2F2 overexpression could abrogate the effect of miR-520a mimics on cell growth by promoting cell cycle progression. Collectively, these results suggest that ANCCA/PRO2000-miR-520a-E2F2 regulatory loop exerts a critical function in promoting cell growth in HCC.

### *ERO1L* and *G3BP2* may be target genes of ANCCA/PRO2000

**Figure 8:**
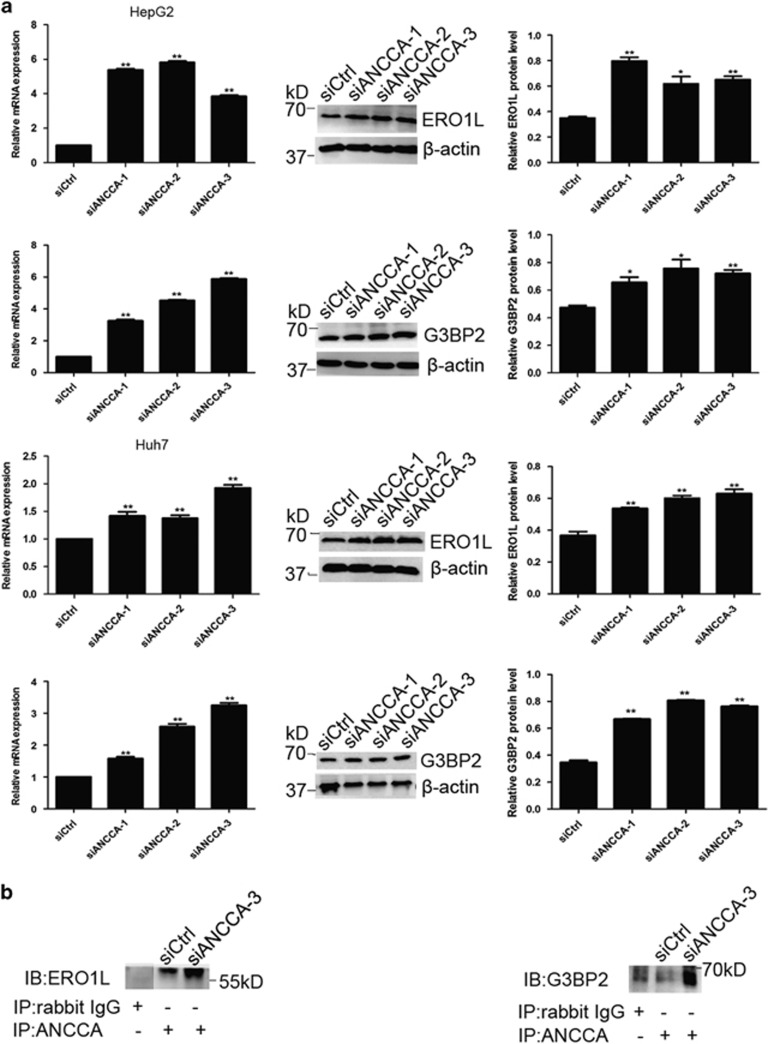
*ERO1L* and *G3BP2* may be target genes of ANCCA/PRO2000. (**a**) qRT–PCR and western blotting validated significant upregulation of ERO1L and G3BP2 expression in siANCCA-treated cells. Data represent mean±s.d. of three independent experiments. (**b**) The association between ANCCA and ERO1L or between ANCCA and G3BP2 was analyzed by co-immunoprecipitation in HepG2 cell. *P<0.05; **P<0.01, versus siCtrl.

Among 25 upregulated genes in the cDNA microarray analysis, *ERO1L* and *G3BP2* were overexpressed for 2.4- and 3.2-fold, respectively. qRT–PCR and western blotting were performed to confirm whether *ERO1L* and *G3BP2* were ANCCA/PRO2000-targeted genes in HCC cells. Compared with protein levels in HepG2 and Huh7 cells transfected with ANCCA-siRNA (Figure 8a).

To examine whether ERO1L and G3BP2 interacts with ANCCA/PRO2000 in HCC cells, we next carried out co-IP assay. As shown in Figure 8b, ERO1L and G3BP2 co-immunoprecipitated with ANCCA/PRO2000 anti-antibody showed a robust increase in siANCCA-treated HepG2 cell, respectively. These findings clearly demonstrate that ANCCA/PRO2000 negatively regulates ERO1L and G3BP2 by, respectively, interacts with them.

### ERO1L and G3BP2 inhibit migration of HCC cells *in vitro*

**Figure 9:**
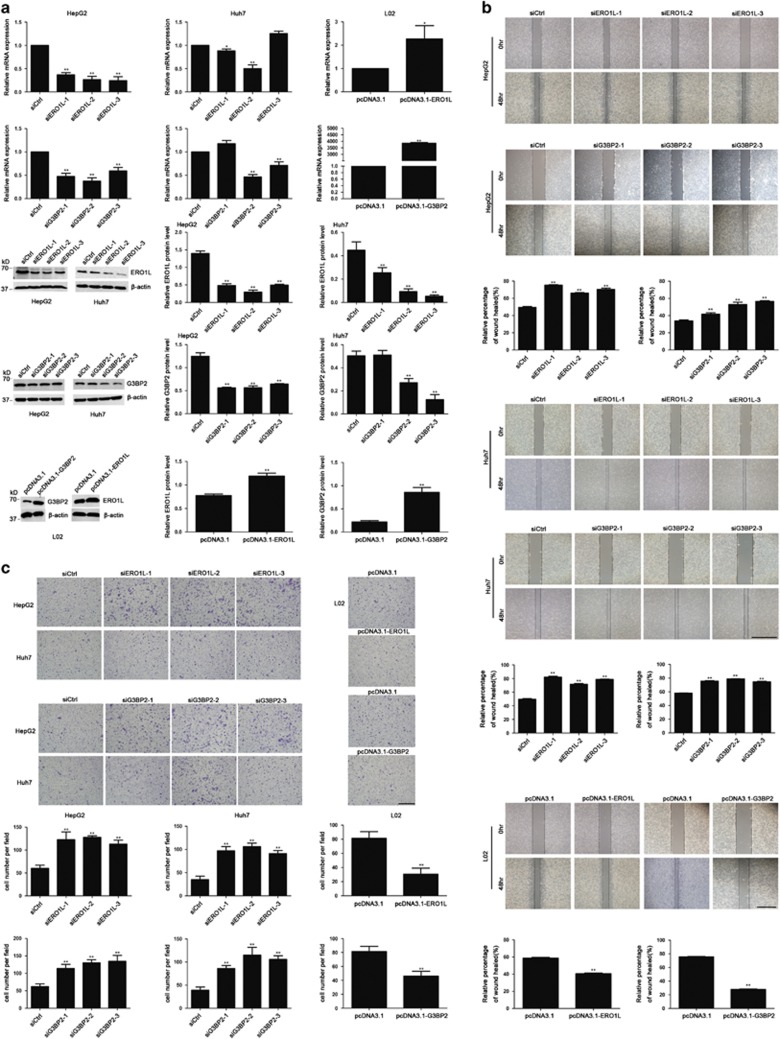
ERO1L/G3BP2 inhibited the migration of HCC cells *in vitro*. (**a**) qRT–PCR and western blotting analyses confirmed that downregulation of ERO1L/G3BP2 in HepG2 and Huh7 cells via siRNA and upregulation in L02 cells by pcDNA3.1-ERO1L/G3BP2. (**b**) Wound-healing assay of HepG2, Huh7 and L02 cells. Cells were imaged 0 and 48 h after scratches were created to measure the percentage of wound-healed area. Representative images at different time points are shown. Scale bar, 500 μm. (**c**) Transwell cell migration assay of HepG2, Huh7 and L02 cells. Representative photographs are showed in the upper panels. Scale bar, 100 um. *P<0.05; **P<0.01, versus siCtrl.

The reverse relationship of ERO1L/G3BP2 and ANCCA/PRO2000 prompted us to further investigate their effects in HCC cells. ERO1L/G3BP2 knockdown with RNA interference (siERO1L and siG3BP2) and upregulation with high-expression plasmid (pcDNA3.1-ERO1L and pcDNA3.1-G3BP2) were attempted in HepG2, Huh7 and L02 cells (Figure 9a). Results of wound-healing assay showed that ERO1L/G3BP2 knockdown by siRNA increased cell motility in HepG2 and Huh7 cells and ERO1l/G3BP2 overexpression by high-expression plasmid decreased cell motility in L02 cell compared with that in control cells (Figure 9b). Similar results were observed in transwell migration assays (Figure 9c). Taken together, these observations indicated that ERO1L/G3BP2 inhibit HCC cell migration.

### ANCCA/PRO2000 is a direct target of miR-372

It is usually accepted that gene expression is regulated by miRNAs. By bioinformatics assay, miR-372/miR-373/miR-106b/miR-93 were predicted to have a potential interaction site at the 3′-UTR of ANCCA/PRO2000 mRNA (Supplementary Figure S1a). To verify whether ANCCA/PRO2000 expression is regulated by these four candidate miRNAs, we transfected the HepG2 and Huh7 cells with the four miRNAs mimics and inhibitors and then measured the level of ANCCA/PRO2000 by qRT–PCR and western blotting. Expression of ANCCA/PRO2000 was decreased after transfection of miR-372 mimics, whereas miR-372 inhibitor did not upregulate the expression of ANCCA/PRO2000 as expected (Supplementary Figures S1c and d). This conflict was attributable to the failure of miR-372 inhibitor to decrease the level of miR-372, which might be explained by the unstability of miRNA inhibitor after transfected into cells (Supplementary Figures S2a and b). Moreover, the viability of HepG2 and Huh7 cells transected with miR-372 mimics significantly decreased compared with that of mimics control-treated cells (Supplementary Figure S1e). The results of luciferase report indicated that miR-372 directly targeted the 3′-UTR of ANCCA/PRO2000 in HCC cells (Supplementary Figure S1b). No significant difference of ANCCA/PRO2000 expression was observed in cells treated with other three miRNAs mimics and inhibitors compared with the controls (Supplementary Figures S2c,b and e).

To further evaluate clinical significance of miR-372 in HCC tissues, we examined miR-372 expression in 46 HCC samples. According to the median value of miR-372 expression, 46 cases of HCC were divided into the miR-372 high-level group (*n*=23) and the miR-372 low-level group (*n*=23). The level of miR-372 expression was significantly downregulated in HCC tissues than that in normal liver tissues (Supplementary Figure S1f). *χ*^2^-test showed that ANCCA/PRO2000 protein was negatively correlated with miRNA-372 expression level (Supplementary Table S3). Moreover, miR-372 level was negatively correlated with tumor number, tumor differentiation, cirrhosis, venous metastasis, TNM stage and recurrence (Supplementary Table S4). Kaplan–Meier analysis showed that HCC patients with high miR-372 level tended to have more favorable prognosis as compared with the low miR-372 level (Supplementary Figure S1g).

## Discussion

**Figure 10:**
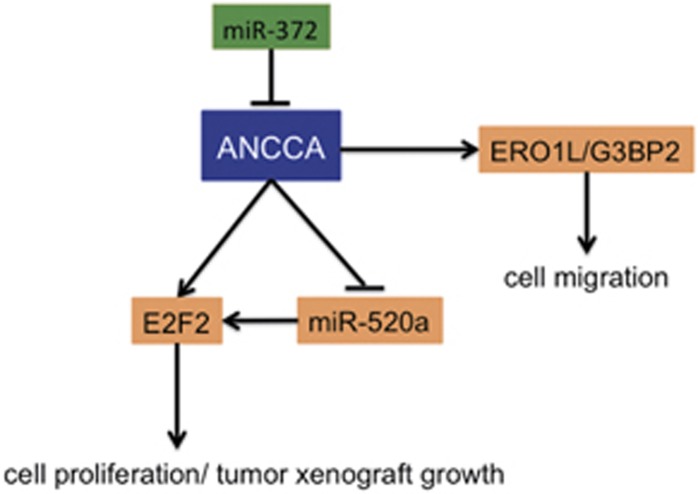
Schematic diagram of the ANCCA/PRO2000-miR-520a-E2F2 regulatory loop in HCC.

In the current work, we disclosed a novel ANCCA/PRO2000-miR-520a-E2F2 regulatory loop that exerts a crucial role in regulating cell proliferation in HCC (Figure 10). More specifically, we found that ANCCA/PRO2000 positively modulates E2F2 expression at both mRNA and protein levels. Furthermore, ANCCA/PRO2000 simultaneously suppresses the expression of miR-520a that could target E2F2, to enhance cell proliferation by promoting cell cycle progression in HCC. This regulatory loop demonstrates a concerted and coordinated mechanism for ANCCA/PRO2000 to regulate cell proliferation in the context of HCC. In addition to cell proliferation, we also presented evidence suggesting that ANCCA/PRO2000 is involved in cell migration partially by negatively regulating ERO1L and G3BP2 expression. Our results elucidated the biological roles and underlying molecular mechanism of ANCCA/PRO2000 and suggested an essential function of ANCCA/PRO2000-miR-520a-E2F2 regulatory loop in the development of HCC.

Previous work from our lab identified that ANCCA/PRO2000 as a proliferation-associated protein predicts poor prognosis in patients with HCC.^17^ Recent studies also suggested that ANCCA/PRO2000 exerts strong oncogenic function based on the investigations on cell proliferation, migration and invasion in HCC.^18, 19^ However, to date, downstream targets and molecular mechanisms related to its oncogenic activity have not been properly explored. We show here that ANCCA/PRO2000 could promote cell proliferation by accelerating cell cycle progression. *In vivo* evidence also demonstrated that ANCCA/PRO2000 inhibition delays tumor xenograft growth, but we have not investigated its effect on mouse survival time. Moreover, by bioinformatics and integrative analytical approaches, ANCCA/PRO2000-associated genes were identified and *E2F2* among them was the only gene implicated in cell cycle regulation. E2F2, as a member of the E2F family of transcription factors, has a crucial role in the control of the G1/S transition and DNA replication in mammalian cells.^33^ Overexpression of E2F2 was detected during c-myc and c-myc/TGFα-induced hepatocarcinogenesis in trans-genetic mice.^34^ In the present study, the deletion of ANCCA/PRO2000 inhibited HCC cell growth and cell cycle progression accompanying with the downregulation of E2F2 expression. To further investigate the potential mechanism how ANCCA/PRO2000 regulates E2F2, we found that ANCCA/PRO2000 interacts with E2F2 through co-IP assay.

Intriguing, our results also revealed that miR-520a, barely studied in HCC, is a crucial mediator between ANCCA/PRO2000 and E2F2. ANCCA/PRO2000 strongly suppressed miR-520a expression as evidenced by a significant increase in miR-520a intracellular levels upon ANCCA/PRO2000 silencing *in vivo* and *in vitro*. Given that extensive evidence revealed that miRNAs have important roles in HCC progression by base-pairing with the 3′-UTR of target mRNAs,^25, 26^ we discovered that E2F2 mRNA may be a target of miR-520a with prediction analysis based on the sequence. Further studies verified that miR-520a negatively modulates E2F2 expression and repressed cell proliferation in an E2F2-dependent manner. The role of E2Fs in digestive malignancies is often dictated by the status of vital cell cycle regulators, such as pRb, p16^INK4A^, with many E2Fs-involved intricate loops.^35, 36^ Thus, the above results revealed that ANCCA/PRO2000-miR-520a-E2F2 regulatory loop cooperatively promoted cell proliferation in HCC cells.

Among ANCCA/PRO2000-associated genes, *ERO1L* and *G3BP2* expressions were negatively correlated with ANCCA/PRO2000. ERO1L, as a hypoxia-inducible endoplasmic reticulum-resident oxidase, has been reported to catalyze the formation of disulfide bond that is critical for protein folding.^37, 38^ Protein misfolding is always involved in cancer, suggesting that ERO1L expression may have a role in cancer. As for G3BP2, it directly associates with the SH3 domain of GTPase-activating protein that functions as an inhibitor of RAS and is closely related to P53.^39, 40^ However, at present, the roles of ERO1L and G3BP2 in HCC are limited. Here we showed that ANCCA/PRO2000 could negatively modulate the expression of both of ERO1L and G3BP2 through interaction with them. Moreover, ERO1L and G3BP2 significantly inhibited HCC cell migration, suggesting that ANCCA/PRO2000 could enhance cell migratory capacity at least partially by negatively regulating ERO1L and G3BP2 expression.

In a previous study, ANCCA/PRO2000 was reported to be the target of miR-372.^18^ In this study, we confirmed the previous finding and further found that overexpression of miR-372 led to a significant reduction in ANCCA/PRO2000 expression and an obvious inhibition of cell proliferation. In addition, the lower miR-372 expression in HCC tissues was demonstrated to significantly correlate with more aggressive tumor behavior and indicated poor prognosis of HCC patients.

In summary, our study elucidated the biological function of ANCCA/PRO2000 in HCC that is in good agreement with previous findings, and we provide a new insight into novel mechanisms for ANCCA/PRO2000 regulating cell proliferation and migration. To our knowledge, this is the first evidence of ANCCA/PRO2000-miR-520a-E2F2 regulatory loop as a driving force for HCC development, and altered components of this regulatory loop may represent attractive therapeutic targets. Certainly, the effects of this loop targeting still need to be evaluated in normal hepatocytes to define its potential therapeutic value more precisely. In addition, the present study provided important clues for investigating the key roles of the noncoding RNA-mediated regulatory network in HCC progression.

## Materials and methods

### Ethics statement

All procedures were performed according to the guidelines of Association for Assessment and Accreditation of Laboratory Animal Care International. This study was approved by the Ethics Committee of The Central South University and all efforts were made to minimize animal suffering and the number of animals used. All of the tissues were the rest of the specimens in pathology department and this study was approved by the Ethics Committee of The Zhujiang Hospital affiliated to Southern Medical University (2013-ZLZX-004). Written informed consent was obtained from all the patients to publish this report.

### Tissue specimens

Archival paraffin-embedded primary HCC samples were obtained from 221 patients by surgical resection in our department between January 2008 and June 2012. Eight normal human liver tissues were obtained from the distal tissue of liver hemangioma. The HCC patients, 190 males (85.97%) and 31 females (14.03%), ranged in age from 25 to 79 years (mean 50). Clinicopathological features of study population were presented in Table 1. All samples were independently reviewed by two pathologists. The cases of HCC were classified according to the criteria described by Edmondso-Steiner^28^ and grouped as well differentiated (grade I–II; *n*=173) or poorly differentiated (grade III–IV; *n*=48). All 221 specimens contained pericarcinomatous tissues, in which including 120 cases with cirrhosis. All tissues were fixed in 10% formalin and embedded in paraffin wax and then 4 μm serial sections were cut. All tissues were fixed in 10% formalin (pH 7.0) for 12–24 h and embedded in paraffin wax and then 4 μm serial sections were cut and mounted on poly-l-lysine-coated slides.

### Cell lines, reagents, antibiotics and antibodies

Huh7, Hep3B, Hep40 and L02 cells were cultured in Dulbecco's modified Eagle medium (DMEM; Gibco, Grand lsland, NY, USA) supplemented with 10% fetal bovine serum (Gibco). HepG2 cells were cultured in Eagle's minimal essential medium (American Type Culture Collection, Manassas, VA, USA) supplemented with 10% fetal bovine serum (Gibco). Cells were maintained at 37 °C in a humidified atmosphere of 5% CO_2_. HepG2 and Huh7 cells stably downexpressing ANCCA/PRO2000 were generated by lentiviral particles of short hairpin RNA targeting ANCCA/PRO2000 mRNA (shANCCA-1, shANCCA-2 and shANCCA-3) infection and puromycin (Invitrogen, Carlsbad, CA, USA) selection. HepG2 and Huh7 cells stably overexpressing E2F2 were generated by transfection with pcDNA3.1-E2F2 and G418 (Merck, Whitehouse Station, NJ, USA) selection. CL48 cells stably with ANCCA/PRO2000 overexpression were generated by transfection with pcDNA3.1-ANCCA/PRO2000 and G418 selection. ANCCA/PRO2000 antiserum was generated by Covance by immunizing rabbits with recombinant ANCCA/PRO2000 N-terminus protein (amino acids 1–264) expressed and purified from *Escherichia coli*. Antibodies specific to E2F2 (sc-633) and β-actin were from Santa Cruz Biotechnology Inc (Santa Cruz, CA, USA). Antibodies specific to ERO1L (ab81959) and G3BP2 (ab86135) were from Abcam Inc (Cambridge, UK).

### Plasmids, oligonucleotides and transfections

The lentiviral particles of shANCCA (shANCCA-1, shANCCA-2 and shANCCA-3) and shCtrl were purchased from GenePharma Inc (Shanghai, China; Supplementary Table S1). The ANCCA/PRO2000 expression plasmid was a gift from HW Chen (UC Davis, Cancer Center, USA). The E2F2, ERO1L, G3BP2 overexpression plasmids (pcDNA3.1-E2F2, pcDNA3.1-ERO1L, pcDNA3.1-G3BP2) were purchased from GeneChem Inc. The siRNA targeting ANCCA/PRO2000 mRNA (siANCCA), ERO1L (siERO1L) and G3BP2 (siG3BP2), siCtrl, miR-372/miR-373/miR-106b/miR-93/miR-520a mimics, inhibitors and negative control were from GenePharma Inc (Supplementary Table S1). Cells were seeded at a density of 2–3 × 10^5^ cells per well in six-well plates with DMEM containing 10% fetal bovine serum for 24 h. Cells in the exponential growth phase were then transfected with the plasmids (2 μg) or oligonucleotides (100 pmol), using Lipofectamine 2000 (Invitrogen) following the manufacturer's protocols.

### Cell proliferation and colony formation assays

Cell proliferation was measured using the CCK-8 assay kit (Dojindo Corporation, Kumamoto, Japan). Cells from stable clones or with transient transfection were plated into each well of a 96-well plate and 10 μl of CCK-8 was added to 90 μl of culture medium at the indicated time. Subsequently, the cells were incubated at 37 °C for 2 h and the optical density was measured at 450 nm. According to the correlation between the absorbance and cell number, cell number was calculated based on the absorbance. For the colony formation assay, cells from stable clones were seeded in triplicates into 60-mm dishes (Corning, Corning, NY, USA) at 800 or 1000 cells per dish and incubated at 37 °C for 2 weeks. Colonies were fixed with methanol, stained with 0.1% crystal violet visualized and then counted under microscopy. For the soft-agar colony formation assay, cells from stable clones were detached from the plates by trypsinization and then were resuspended as individuals in DMEM growth medium with the full supplements mixed at a 3:1 ratio with 1.6% agarose (Amresco, Solon, OH, USA). The mixture was then plated onto six-well plates at 500 cells per well over a bottom layer of 0.8% agarose in DMEM with the supplements. Cells were maintained at 37 °C with a medium change every 3 days. Three weeks later, cell aggregates with diameters of 0.2 mm or larger (containing ~50 or more cells) were counted as colonies.

### Cell migration and invasion assays

Wound-healing and transwell migration assays were performed to evaluate cell migration in HepG2 and Huh7 cells with transient transfection and L02 cells from stable clones. When cultured cells reached 90% confluence, they were scratched with a pipette tip and cultured in serum-free medium. Recovery of the disruption was observed for 0, 24 and 48 h. For transwell migration assay, cells were plated on the upper surface of the transwell chamber (Corning) and incubated for 24 h. The migrated cells at the lower surface of the chamber were fixed with 75% methanol and stained with crystal violet (Sigma, St Louis, MO, USA). Invasion assays were observed using a modified transwell chamber system (BD Biosciences, San Jose, CA, USA) according the manufacturer's protocols. Cells from stable clones were seeded onto matrigel-coated membrane inserts with a pore size of 8 μm. Medium containing 10% fetal bovine serum served as the chemoattractant in the lower chamber. The invasive cells attached to the lower surface of the chamber after 48-h incubation were fixed with 75% methanol and stained with 0.1% crystal violet, and counted under a microscope.

### Cell cycle analysis

Cells were collected and fixed in 70% ethanol at 4 °C for 16 h and then stained with propidium iodide. The distribution of cells within each of the cell cycle compartments was measured using a Becton-Dickinson FACS Calibur machine (San Jose, CA, USA).

### RNA extraction and reverse transcription

Total RNA was prepared using the RNeasy Mini Kit (Qiagen, Germantown, MD, USA) following the manufacturer's protocol. For formalin-fixed paraffin-embedded samples, total RNA was extracted from 5–20 10-μm-thick sections using miRNeasy FFPE Kit (Qiagen) according to the manufacturer's instructions. The quantity and quality of the total RNA were evaluated using the NanoDrop spectrophotometer (Thermo Fisher Scientific Incorporated, Waltham, MA, USA) according to the manufacturer's instructions. An amount of 1 μg of total RNA from each sample was reverse-transcribed to cDNA with PrimeScript reverse transcriptase reagent kit (TaKaRa, Dalian, China) according to the manufacturer's instructions.

### Real-time qRT–PCR of mRNA and miRNA

Quantitative real-time PCR was carried out in ABI Prism 7500 Sequence Detection System (Applied Biosystems, Foster City, CA, USA) using SYBR Premix Ex Taq (TaKaRa) according to the manufacturer's instructions. The miRNA sequence-specific RT–PCR for miR-372/miR-373/miR-106b/miR-93/miR-520a and endogenous control U6 was performed according to Hairpin-it miRNAs, qPCR quantitation kit and U6 snRNA real-time PCR normalization kit (GenePharma). All qPCR reactions, including no-template controls, were performed in triplicate. Expression levels of each mRNA or miRNA were evaluated using comparative threshold cycle (Ct) method as normalized to that of GAPDH or U6 (2^−ΔΔCT^). The sequences for primers were provided in Supplementary Table S2.

### Western blotting and co-IP

Total protein was extracted from cells in the logarithmic growth phase. Cells were lysed and sonicated in a solution containing 0.5% sodium deoxycholate (w/v), 0.2% SDS (w/v), 1% Triton X-100 (v/v), 5 mm EDTA, 10 mg/ml leupeptin, 10 mg/ml aprotinin and 1 mm phenylmethyl sulfonyl–uoride supplemented with 1:1000 dilution of protease inhibitor cocktail. The homogenates were then centrifuged at 4 °C for 10 min to remove cell debris. Supernatants were collected and concentrations were determined by the DC protein assay (Bio-Rad Laboratories, Hercules, CA, USA). Equivalent amounts of protein were electrophoresed on an 8% polyacrylamide gel and then transferred to a polyvinylidene difluoride membrane. For western blotting, the membrane was blocked with 5% milk (w/v) in TBS containing 0.1% Tween 20 (TBS/T) at room temperature for 2 h and incubated with primary antibodies including ANCCA/PRO2000 (1:1000), E2F2 (1:1000), ERO1L (1:1000) and G3BP2 (1:2000) overnight at 4 °C, washed three times with TBST, followed by incubation with appropriate secondary antibodies at room temperature for 1 h. The immune complexes were detected by an enhanced chemiluminescence system (Amersham, Arlington Heights, IL, USA). For co-IP, protein extracts (0.2 mg) were incubated with anti-E2F2, anti-ERO1L or anti-G3BP2 antibody overnight at 4 °C. Subsequently, antibodies were collected with protein A-/protein G-Sepharose beads, and protein complexes were washed three times at 4 °C with the lysis buffer, electrophoresed on an SDS–polyacrylamide gel under reducing conditions, and transferred onto polyvinylidene difluoride membrane. Immunoblotting was incubated with the indicated primary antibody ANCCA/PRO2000 overnight at 4 °C and followed by incubation with appropriate horseradish peroxidase-conjugated secondary antibody at room temperature for 1 h, and developed with enhanced chemiluminescence reagents.

### cDNA expression array

Human genome oligo array (22 K) was designed by CapitalBio Corporation (Beijing, China). CapitalBio 22 K Human Genome Oligo Array comprises 21 522 70-mer oligo probes, each representing one transcript of the human genome. RNA samples as prepared above were labeled with Cy5 (red) and Cy3 (green) dyes, respectively. Labeled samples were then hybridized to array gene chips. Arrays were scanned using CapitalBio's confocal scanner LuxScan 10 K-A (Beijing, China) and the obtained images were analyzed with SpotData software (CapitalBio). An intensity-dependent lowess program in the R language package was used to normalize the two channel ratio values.

### 3′-UTR luciferase reporter assay

For luciferase reporter experiments, the wild and mutational 3′-UTR segments of ANCCA/PRO2000 predicted to interact with miR-372 were amplified by PCR from human genomic DNA and inserted into psiCHECK2 vector immediately downstream from the stop codon of luciferase (Promega, Madison, WI, USA) to develop psiCHECK2-ANCCA/PRO2000-3′UTR and psiCHECK2-ANCCA/PRO2000-mut-3′UTR. HepG2 cells were transfected with 0.4 μg of the luciferase reporter psiCHECK2 control, 0.5 μg psiCHECK2-ANCCA/PRO2000-3′UTR constructs or psiCHECK2-ANCCA/PRO2000-mut-3′UTR. Co-transfection of miR-372 mimics or inhibitors at 100 nm was also performed in HepG2 cells. Luciferase activity was measured by the dual-luciferase assays (Promega) and the data were showed in Supplementary Figure S1.

### *In vivo* tumor xenograft model

Female BALB/c nude mice aged 6–8 weeks were purchased from the Guangdong Laboratory Animal Center, China (National certification No. 2006A015). All procedures were performed according to the guidelines of Association for Assessment and Accreditation of Laboratory Animal Care International. HepG2, Huh7 or L02 cells from stable clones were collected, washed with PBS and resuspended in serum-free medium at a concentration of 1 × 10^7^ cells per 0.2 ml. Twenty-four mice were randomly divided into three groups for HepG2 (shANCCA/Ctrl), Huh7 (shANCCA/Ctrl) and L02 (pcDNA3.1-ANCCA/pcDNA3.1) cells. We were single-blinded to the group allocation. Each subculturing situs was injected with viable 4 × 10^6^ per 0.2 ml of HepG2 or Huh7 and 6 × 10^6^ per 0.2 ml of L02 in the back leg to establish the cancer model, respectively. Tumor size was monitored every 3 days, and mice were killed after 4–5 weeks. The tumors were excised and measured. The tumor volume (*V*) was calculated using the following equation: *V*=1/2 × *S*^2^ × *L*, where *S* and *L* are the shortest and longest diameter of the tumor, respectively.

### Immunohistochemical staining

Sections were deparaffinized and rehydrated routinely. Before adding the primary antibody, antigen was retrieved by heating sections in 10 mm citrate buffer (pH 6.0) in a microwave oven for 10 min, followed by 10 min of cooling. After blocking with 0.3% H_2_O_2_ and goat serum, the slides were then incubated with a primary antibody, directed against ANCCA/PRO2000 (1:100 dilution) and E2F2 (1:50 dilution; Santa Cruz Biotechnology Inc) at 4 °C overnight. Biotinylated secondary antibodies were then applied according to the manufacturer's recommendations (Amersham). After incubation with avidin–biotin complex using the Vector Elite ABC detection kit (Vector Labs, Burlingame, CA, USA), reaction products were visualized by 3-diaminobenzidine, and the slides were subsequently counterstained with hematoxylin. Brown-yellow granules in the nucleus or cytoplasm were considered positive staining. The positive reactivity was scored semiquantitatively by microscopic evaluation according to the estimated number of positive nuclei or cytoplasm of target cells. The scores were graded into four groups from 0 to +3 as follow: 0, no positive cells; +1, <25% of positive cells; +2, 25–50% of positive cells; and +3, >50% of positive cells. Negative controls were performed by replacing the primary antibodies stated above with PBS. Image quantitative analysis was performed using Image ProPlus 6 AMS software (Media Cybernetics Inc, Buckinghamshire, UK). ANCCA/PRO2000 and E2F2 expression intensity was assessed by estimating the integrated optical density.

### Statistical analysis

Data are represented as mean±s.d. Possible differences between the groups were analyzed using independent samples *T-*test or one-way analysis of variance. The association between ANCCA/PRO2000 expression and clinical features were analyzed by Pearson *χ*^2^-test. Survival curves were obtained by Kaplain–Meier analysis. Prognostic factors were examined by univariate and multivariate analyses (Cox proportional hazards model). A *P-*value <0.05 was considered significant. All statistical analyses were carried out using SPSS 17.0 software (SPSS Inc., Chicago, IL, USA).

## Figures and Tables

**Table 1:** Correlation of ANCCA expression with clinicopathological variables of 221 cases of HCC

*Variables*	*Cases*	*ANCCA protein expression*			P*-value*
		*−*	*+*	*++*	
Gender					0.265
Male	190	64	105	21	
Female	31	15	14	2	
					
Age (years)					0.549
⩽50	107	40	54	13	
>50	114	39	65	10	
					
Serum HBsAg					0.857
Negative	104	39	54	11	
Positive	117	40	65	12	
					
Serum AFP (ng/ml)					0.979
<20	102	36	55	11	
>20	118	43	63	12	
					
Tumor size (cm)					0.020
⩽5	109	41	63	5	
>5	112	38	56	18	
					
Tumor number					<0.001
Single	169	76	78	15	
Multiple	52	3	41	8	
					
Tumor differentiation					<0.001
I–II	173	76	93	4	
III–IV	48	3	26	19	
					
Cirrhosis					0.018
Absent	101	45	44	12	
Present	120	34	75	11	
					
TNM stage					<0.001
I–II	171	78	87	6	
III–IV	50	1	32	17	
					
Tumor microsatellite					<0.001
Absent	167	76	76	15	
Present	54	3	43	8	
					
Portal vein tumor thrombus					<0.001
Absent	197	79	104	14	
Present	24	0	15	9	
					
Recurrence					<0.001
Absent	198	78	106	14	
Present	23	1	13	9	

*−*, negative; +, moderate positive; ++, strong positive.

The *P*-value was calculated by Pearson *χ*^2^-test.

**Table 2:** Univariate and multivariate analyses of potential prognostic factors associated with overall survival of HCC patients

*Variables*	*Cases*	*Univariate analysis*		*Multivariate analysis*	
		*HR (95% CI)*	P*-value*	*HR (95% CI)*	P*-value*
Gender (male/female)	190/31	0.701 (0.404–1.216)	0.207		
Age (⩽50/>50 years)	107/114	2.475 (1.588–3.858)	<0.001	2.621 (1.661–4.135)	<0.001
Serum HBsAg (negative/positive)	104/117	1.022 (0.690–1.514)	0.913		
Serum AFP (⩽20/>20 ng/ml)	102/118	0.897 (0.606–1.327)	0.587		
Tumor size (⩽5/>5 cm)	109/112	1.288 (0.868–1.911)	0.209		
Tumor number (single/multiple)	169/52	1.849 (1.168–2.929)	0.009	2.346 (0.313–17.575)	0.407
Tumor differentiation (I–II/III–IV)	173/48	7.228 (4.450–11.742)	<0.001	1.988 (1.090–3.623)	0.025
Cirrhosis (absent/present)	101/120	1.069 (0.720–1.589)	0.740		
TNM stage (I–II/III–IV)	171/50	10.935 (6.645–17.995)	<0.001	7.483 (3.978–14.074)	<0.001
Tumor microsatellite (absent/present)	167/54	1.809 (1.137–2.877)	0.012	0.399 (0.051–3.133)	0.382
Portal vein tumor thrombus (absent/present)	197/24	5.518 (3.189–9.549)	<0.001	0.811 (0.408–1.613)	0.551
Recurrence (absent/present)	198/23	5.222 (2.608–10.454)	<0.001	1.011 (0.484–2.109)	0.977
ANCCA expression (−/+/++)	79/119/23	6.955 (4.604–10.508)	<0.001	4.507 (2.697–7.534)	<0.001
MiR-372 expression (low/high)	23/23	0.015 (0.000–10.927)	0.212		

CI, confidence interval; HCC, hepatocellular carcinoma; HR, hazard ratio.

The *P*-value was calculated by Cox proportional hazards model.
