# Supplementary figures and images for: An ANCCA/PRO2000-miR-520a-E2F2 regulatory loop as a driving force for the development of hepatocellular carcinoma
Source: Oncogenesis. 2016 May 30;5(5):e229–. doi: 10.1038/oncsis.2016.22 (PMC4945746; doi:10.1038/oncsis.2016.22)

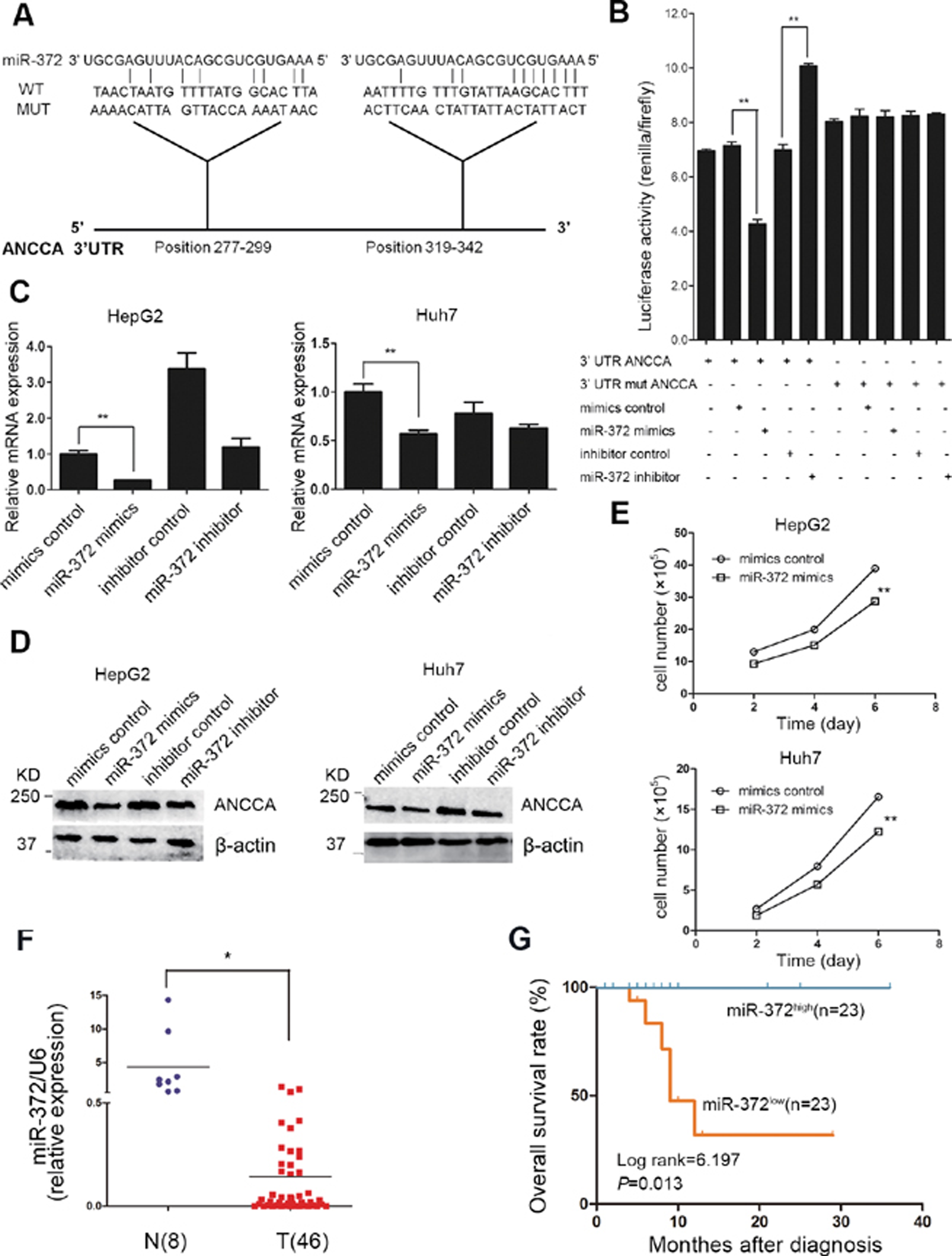

Supplement: Supplementary Figure S1 [file oncsis201622x1.tif]

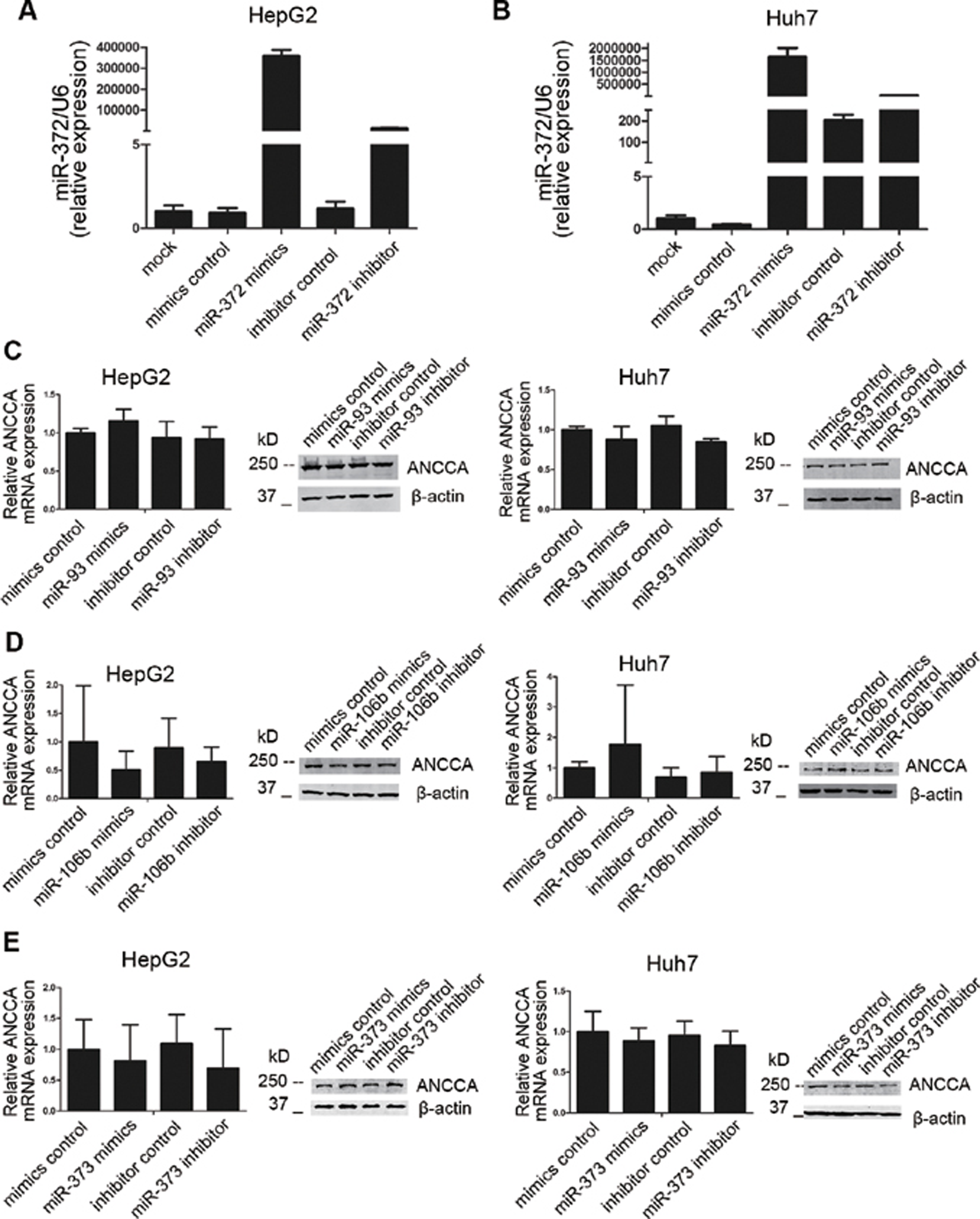

Supplement: Supplementary Figure S2 [file oncsis201622x2.tif]
