# Supplementary material for: An ANCCA/PRO2000-miR-520a-E2F2 regulatory loop as a driving force for the development of hepatocellular carcinoma
Source: Oncogenesis. 2016 May 30;5(5):e229–. doi: 10.1038/oncsis.2016.22 (PMC4945746; doi:10.1038/oncsis.2016.22)
Supplement: Supplementary Figure Legends [file oncsis201622x3.docx]

**Supplementary Figure legends**

**Figure S1**. miR-372 directly target ANCCA/PRO2000. (A) Predicted binding sites of miR-372 in ANCCA/PRO2000 3’-UTR and the sites of target mutagenesis. (B) Dual-luciferase assays showing a decrease or an increase in luciferase activity by cotransfecting wild-type UTR and miR-372 mimics or inhibitor. Values are shown as luciferase expression ratio of renilla to firefly. (C-D) ANCCA/PRO2000 mRNA and protein levels were assessed after transfection of miR-372 mimics, inhibitor and corresponding controls in HepG2 and Huh7cells. (E) Cell growth curves of HepG2 and Huh7 cells after transfected with miR-372 mimics and control. (F) The expression levels of miR-372 in HCC tissues (T, n=8) and adjacent nontumor tissues (N, n=46). (G) Kaplan–Meier analysis of overall survival of 51 patients with HCC based on miR-372 expression. *, *P*<0.05; **, *P*<0.01.

**Figure S2**. The expression levels of miR-372 and effects of miR-93, miR-106b, miR-373 mimics and inhibitors in HepG2and Huh7 cells. (A) MiR-372 mRNA level was analysed by qRT-PCT in HepG2 cells. (B) MiR-372 mRNA level was analysed by qRT-PCT in Huh7 cells. (C-E) ANCCA/PRO2000 mRNA and protein levels were assessed after transfection of miR-93/miR-106b/ miR-373 mimics, inhibitor and corresponding controls in HepG2 and Huh7 cells.
