# Supplementary material for: An ANCCA/PRO2000-miR-520a-E2F2 regulatory loop as a driving force for the development of hepatocellular carcinoma
Source: Oncogenesis. 2016 May 30;5(5):e229–. doi: 10.1038/oncsis.2016.22 (PMC4945746; doi:10.1038/oncsis.2016.22)
Supplement: Supplementary Tables [file oncsis201622x4.docx]

**Supplementary Tables**

**Supplementary Table 1. Sequences for RNAs oligo used in the study**

| **Name** | **Sense sequence (5’-3’)** | **Antisense sequence (5’-3’)** |
| --- | --- | --- |
| **siCtrl** | UUCUCCGAACGUGUCACGUTT | ACGUGACACGUUCGGAGAATT |
| **siANCCA-1** | GGGCUAGAAACAUCGUUCATT | UGAACGAUGUUUCUAGCCCTT |
| **siANCCA-2** | GCUACUGUUUACUAUCAGGCUTT | AGCCUGAUAGUAAACAGUAGCTT |
| **siANCCA-3** | GGACCAAGAAGUCCUUACUTT | AGUAAGGACUUCUUGGUCCTT |
| **siERO1L-1** | CGUGUCCUUUCUGGAAUGATT | UCAUUCCAGAAAGGACACGTT |
| **siERO1L-2** | GUGCAUUUGAGUGCAAGAUTT | AUCUUGCACUCAAAUGCACTT |
| **siERO1L-3** | UAACUCUUUACUGGAAAUATT | UAUUUCCAGUAAAGAGUUGTT |
| **siG3BP2-1** | GCUCCGGAAUAUUUACACATT | UGUGUAAAUAUUCCGGAGCTT |
| **siG3BP2-2** | CAGUGAAUGUCAUACUAAATT | UUUAGUAUGACAUUCACUGTT |
| **siG3BP2-3** | GAUGAUCGCAGGGAUAUUATT | UAAUAUCCCUGCGAUCAUCTT |
| **shCtrl** | TTCTCCGAACGTGTCACGTTTC |  |
| **shANCCA-1** | GCTGCTAAGCCTCCTATATCA |  |
| **shANCCA-2** | GCATAGAGCCTGTGCTTTAAG |  |
| **shANCCA-3** | GCTTGTAATGGAGATGCTTCT |  |
| **has-mir-372 mimics** | AAAGUGCUGCGACAUUUGAGCGU | GCUCAAAUGUCGCAGCACUUUUU |
| **has-mir-372 inhibitor** | ACGCUCAAAUGUCGCAGCACUUU |  |
| **has-mir-520a-3p mimics** | AAAGUGCUUCCCUUUGGACUGU | AGUCCAAAGGGAAGCACUUUUU |
| **Mimics control** | UUCUCCGAACGUGUCACGUTT | ACGUGACACGUUCGGAGAATT |
| **Inhibitor control** | CAGUACUUUUGUGUAGUACAA |  |

**Supplementary Table 2. Sequences for primers used in the study**

| **Name** | **Sense sequence (5’-3’)** | **Antisense sequence (5’-3’)** |
| --- | --- | --- |
| **ANCCA** | CACCGAGTACTCCTGTGGCTTG | TCTAGCTCGAGTCATTCGCAGAACAC |
| **E2F2** | CGTCCCTGAGTTCCCAACC | GCGAAGTGTCATACCGAGTCTT |
| **ERO1L** | ATTTCCTTTGCATTTTGATGA | TGAAATTCCACTCTTT CGCC |
| **G3BP2** | CACCAGTCTCACAGCCAAGA | CCAGGTCGTTCTCTAGGTCG |
| **GAPDH** | AGAAGGCTGGGGCTCATTTG | AGGGGCCATCCACAGTCTTC |

**Supplementary Table 3. Correlation between ANCCA expression and the levels of miR-372 in 46 cases of HCC**

| ANCCA expression | MiR-372 expression | | *P* |
| --- | --- | --- | --- |
|  | Low | High |  |
| - | 1 | 14 | <0.001 |
| + | 11 | 8 |  |
| ++ | 11 | 1 |  |

-, negative; +, moderate positive; ++, strong positive. The P-value was calculated by Pearson Chi-Square test.

**Supplementary Table 4. Correlation of the level of miR-372 with clinicopathological variables of 46 cases of HCC**

| **Variables** | **cases** | **MiR-372 expression** | | ***P*** |
| --- | --- | --- | --- | --- |
|  |  | **Low** | **High** |  |
| **Gender** |  |  |  | 1.000 |
| Male | 40 | 20 | 20 |  |
| Female | 6 | 3 | 3 |  |
| **Age (years)** |  |  |  | 1.000 |
| ≤48 | 24 | 12 | 12 |  |
| >48 | 22 | 11 | 11 |  |
| **Serum HBsAg** |  |  |  | 0.345 |
| Negative | 15 | 9 | 6 |  |
| Positive | 31 | 14 | 17 |  |
| **Serum AFP**  **(ng/ml)** |  |  |  | 0.376 |
| <20 | 23 | 10 | 13 |  |
| >20 | 23 | 13 | 10 |  |
| **Tumor size**  **(cm)** |  |  |  | 0.555 |
| ≤5 | 22 | 10 | 12 |  |
| >5 | 24 | 13 | 11 |  |
| **Tumor number** |  |  |  | 0.022 |
| Single | 33 | 13 | 20 |  |
| Multiple | 13 | 10 | 3 |  |
| **Tumor differentiation** |  |  |  | <0.001 |
| Ⅰ-Ⅱ | 28 | 8 | 20 |  |
| Ⅲ-Ⅳ | 18 | 15 | 3 |  |
| **Cirrhosis** |  |  |  | 0.018 |
| Absent | 24 | 8 | 16 |  |
| Present | 22 | 15 | 7 |  |
| **TNM stage** |  |  |  | <0.001 |
| Ⅰ-Ⅱ | 29 | 6 | 23 |  |
| Ⅲ-Ⅳ | 17 | 17 | 0 |  |
| **Tumor microsatellite** |  |  |  | 0.063 |
| Absent | 30 | 12 | 18 |  |
| Present | 16 | 11 | 5 |  |
| **Portal vein tumor thrombus** |  |  |  | 0.004 |
| Absent | 39 | 16 | 23 |  |
| Present | 7 | 7 | 0 |  |
| **Recurrence** |  |  |  | 0.007 |
| Absent | 34 | 13 | 21 |  |
| Present | 12 | 10 | 2 |  |

The P-value was calculated by Pearson Chi-Square test.
